# The Relationship between Phenolics and Flavonoids Production with Total Non Structural Carbohydrate and Photosynthetic Rate in *Labisia pumila* Benth. under High CO_2_ and Nitrogen Fertilization

**DOI:** 10.3390/molecules16010162

**Published:** 2010-12-29

**Authors:** Mohd Hafiz Ibrahim, Hawa Z.E. Jaafar, Asmah Rahmat, Zaharah Abdul Rahman

**Affiliations:** 1 Department of Crop Science, Faculty of Agriculture, University Putra Malaysia, 43400 Serdang, Selangor, Malaysia; E-Mail: mhafizphd@gmail.com (M.H.I.); 2 Department of Nutrition & Dietetics, Faculty of Medicine & Health Sciences, University Putra Malaysia, 43400 Serdang, Selangor, Malaysia; E-Mail: asmah@medic.upm.edu.my; 3 Department of Land Management, Faculty of Agriculture, University Putra Malaysia, 43400 Serdang, Selangor, Malaysia; E-Mail: zaharah@agri.upm.edu.my

**Keywords:** CO_2_ enrichment, total phenolics and flavonoids, carbon:nitrogen ratio, photosynthesis nitrogen use efficiency, total soluble sugar and starch profiling, Kacip Fatimah, medicinal herb

## Abstract

A factorial split plot 4 × 3 experiment was designed to examine and characterize the relationship among production of secondary metabolites (total phenolics, TP; total flavonoids, TF), carbohydrate content and photosynthesis of three varieties of the Malaysian medicinal herb *Labisia pumila* Benth. namely the varieties *alata*, *pumila* and *lanceolata* under CO_2_ enrichment (1,200 µmol mol^-1^) combined with four levels of nitrogen fertilization (0, 90, 180 and 270 kg N ha^-1^). No varietal differences were observed, however, as the levels of nitrogen increased from 0 to 270 kg N ha^-1^, the production of TP and TF decreased in the order leaves>roots>stems. The production of TP and TF was related to increased total non structural carbohydrate (TNC), where the increase in starch content was larger than that in sugar concentration. Nevertheless, the regression analysis exhibited a higher influence of soluble sugar concentration (r^2^ = 0.88) than starch on TP and TF biosynthesis. Photosynthesis, on the other hand, displayed a significant negative relationship with TP and TF production (r^2^ = -0.87). A decrease in photosynthetic rate with increasing secondary metabolites might be due to an increase in the shikimic acid pathway that results in enhanced production of TP and TF. Chlorophyll content exhibited very significant negative relationships with total soluble sugar, starch and total non structural carbohydrate.

## 1. Introduction

*Labisia pumila* Benth., popularly known as Kacip Fatimah, is a sub-herbaceous plant with creeping stems from the family Myrsinaceae that is found widespread in Indochina and throughout the Malaysian forest [1]. Traditionally *L. pumila* has been used by Malay women to induce and facilitate childbirth as well as a post-partum medicine [2]. Stone [3] had categorized three varieties of this herb in Malaysia, namely *L. pumila* var. *alata*, *L. pumila* var. *pumila* and *L. pumila* var. *lanceolata*. Each of the varieties has a different usage. The varieties most universally utilized by the traditional healers are the first two, *L. pumila* var. *alata* and *L. pumila* var *pumila.* The other uses of this herb are treatment for dysentery, dysmenorrheal, flatulence, and gonorrhea treatments [4].

Recently, it was found that the bioactive compounds of *L. pumila* consisted of resorcinols, flavonoids and phenolic acid [1,5]. These compounds have been identified as natural antioxidants that may reduce oxidative damage to the human body [6]. The concentration of plant secondary metabolites was found to be influenced by environmental conditions such as light intensity, carbon dioxide levels, temperature, fertilization, biotic and abiotic factors, which can change the concentration of these active constituents [1,7]. Lately, it was found that the enrichment of *L*. *pumila* with high levels of CO_2_ increased the secondary metabolite production (phenolics and flavonoids) of this plant [8]. A similar result was also observed in ginger (Gingiber officianale) [9].

The increase in atmospheric CO_2_ due to climate change has direct effects on plant secondary metabolites. The effects show a wide range of patterns, either in the amounts of primary and secondary metabolites. Plants produce a wide range of carbon-based secondary metabolites (CBSM) which have important functions such as wound healing, defense against herbivores, control of the rates of plant decomposition and mediation of interaction between plants and soil biota [10]. Among these CBSM the polyphehols derived from the phenylpropanoid pathways such as soluble phenolics and flavonoids are quantitatively the most important, accounting for about 30% of the organic carbon cycling in the terrestrial biosphere [11]. Under optimum CO_2_ concentration conditions combined with nutrient resource limitation, which restrict growths to a greater extent than photosynthesis, plants showed an increase in the C/N ratios and excess of non-structural carbohydrates [12]. This excess may be then available for the incorporation in CBSM. The carbon nutrient balance (CNB) hypothesis predicts that the availability of excess carbon at a certain nutrient levels leads to the increased production of CBSM metabolites and their precursors [13]. It was also noted that nitrogen fertilization was found to decrease levels of soluble phenolics and condensed tannins in plant tissues [14]. 

It has been shown that trees grown under elevated CO_2_ concentrations tend to increase photosynthesis and decrease nitrogen concentration relative to biomass [15]. The increase in plant productivity in response to rising CO_2_ is largely dictated by photosynthesis, respiration, carbohydrate production and their differential allocation between plant organs and the subsequent incorporation into biomass. For this reason, many studies have investigated the effects of elevated CO_2_ on plant primary metabolism but relatively few studies have investigated the response of plant CBSM to increasing CO_2_ and its interaction with nitrogen availability. 

The objective of this study was to examine the effects of different nitrogen levels under CO_2_ enrichment on photosynthesis rate, photosynthesis-nitrogen use efficiency (PNUE), C/N ratio, chlorophyll content, primary (total non structural carbohydrate) and secondary metabolite (flavonoids and phenolics) synthesis in three varieties of *L. pumila*. The relationships among photosynthesis, carbohydrate, and total phenolics and flavonoids of plants exposed to combined CO_2_ enrichment and nitrogen levels were also determined.

## 2. Results and Discussion

### 2.1. Total Flavonoids and Phenolics Content and Their Profiling

Nitrogen levels had a significant (*P* ≤ 0.05) impact on the production of total phenolics and flavonoid production (Table 1). As more nitrogen was invested steadily from 0 to 270 kg N ha^-1^, less total phenolic and flavonoid was produced. *Labisia pumila* Benth. partitioning more of their secondary metabolites in the leaves, followed by the roots and then stems. 

**Table 1:** Accumulation and partitioning of total flavonoids (TF) and total phenolics (TP) in different plant parts of *Labisia pumila* Benth. under different combinations of nitrogen levels and CO_2_ enrichment.

Nitrogen levels	Plant parts	TF (mg Rutin/g dry weight)	TP (mg Gallic acid/g dry weight)
A-180 kg N ha^-1^*	Leaf	0.317 ± 0.017c	0.581 ± 0.019d
	Stem	0.143 ± 0.017d	0.301 ± 0.017d
	Root	0.308 ± 0.015c	0.296 ± 0.013d
0 kg N ha^-1^	Leaf	0.803 ± r0.013a	1.410 ± r0.028a
	Stem	0.678 ± r0.022a	1.164 ± r0.029a
	Root	0.788 ± r0.013a	1.222 ± r0.039a
90 kg N ha^-1^	Leaf	0.515 ± r0.022b	1.180 ± r0.032b
	Stem	0.388 ± r0.030b	0.927 ± r0.037b
	Root	0.498 ± r0.022b	0.983 ± r0.051b
180 kg N ha^-1^	Leaf	0.480 ± r0.013c	0.862 ± r0.021c
	Stem	0.357 ± r0.010c	0.610 ± r0.025c
	Root	0.464 ± r0.015c	0.666 ± r0.040c
270 kg N ha^-1^	Leaf	0.212 ± r0.025d	0.576 ± r0.008d
	Stem	0.116 ± r0.023d	0.324 ± r0.011d
	Root	0.197 ± r0.026d	0.380 ± r0.028d

All analyses are mean ± standard error of mean (SEM), N = 15. Means not sharing a common single letter were significantly different at P ≤ 0.05. A = Control at ambient CO_2_ levels (400 µmol^-1^ mol^-1^) and standard Nitrogen fertilization rates (180 kg N ha^-1^).

The enhancement of total flavonoids and phenolics of *L. pumila* seedling was higher under elevated CO_2_ compared to ambient; and when combined with nitrogen at 180 kg ha^-1^, total flavonoids and phenolics increased by 70% and 170%, respectively. The enhancement of total plant flavonoids and phenolics usually occurred when plant is deficient in nitrogen [16,17]. This improvement in plant secondary metabolites might be due to increased total non structural carbohydrates (TNC), as exhibited by the correlation coefficient (r^2^ = 0.81) in Table 2, although a higher correlation coefficient (r^2^ = 0.88) was displayed by total soluble sugar implying that the accumulation of soluble sugar might be more responsible in the up regulation of plant secondary metabolites production. Amin *et al.* [18] had proposed that the increase in flavonoids content was due to increase in total soluble sugar as observed in onion the increase of in the former by 7% as a result of the latter’s enhancement by 21%.

**Table 2:** Correlations among the measured parameters in the experiments.

Characteristics	1	2	3	4	5	6	7	8	9	10	11	12
1. Photosynthesis	1											
2. PNUE	0.04	1										
3. Nitrogen	0.85*	-0.47	1									
4. C:N ratio	-0.73*	0.55	-0.91**	1								
5. Chlorophyll a	0.77**	-0.38	0.88**	-0.81**	1							
6. Chlorophyll b	0.77**	-0.38	0.88**	-0.81**	0.90	1						
7. T. Chlorophyll	0.77*	-0.38	0.88*	-0.81*	0.90	0.90	1					
8. TSS	-0.69*	0.43	-0.83**	0.81**	-0.88**	-0.88**	-0.88**	1				
9. Starch	-0.48	0.26	-0.55**	0.57**	-0.72**	-0.72**	-0.72**	0.88*	1			
10. TNC	-0.58*	0.34	-0.69*	0.69*	-0.81*	-0.81*	-0.81*	0.96**	0.98**	1		
11. Flavonoids	0.77**	-0.38	0.88**	-0.81*	0.90**	0.90**	0.90**	-0.88*	-0.72**	-0.80**	1	
12. Phenolics	0.77**	-0.38*	0.88**	-0.81*	0.90**	0.90**	0.90**	-0.88*	-0.72*	-0.81*	1.00	1

* and ** respectively significant at *P* ≤ 0.05 or *P* ≤ 0.01.

### 2.2. Total Soluble Sugar, Starch and Total Non Structural Carbohydrate (TNC) and Their Profiling

The accumulation and partitioning of carbohydrates were influenced by the nitrogen levels applied to *L. pumila* (P ≤ 0.05). The accumulation of carbohydrates in different parts of the plant followed a descending order of leaf>root>stem. As the nitrogen fertilization increased, the concentration of total soluble sugar, starch and TNC decreased (Table 3). The concentration of sucrose and starch registered the lowest values under 270 kg N ha^-1^, compared to other nitrogen treatments. Under ambient conditions at a standard fertilization rate of 180 kg N ha^-1^ less sucrose and starch were produced in the leaf, stem and root compared to those plants exposed to high CO_2_ concentration with the same fertilization level. In all plant parts of *L. pumila*, the increase in starch content was larger than the increase in sugar concentration [19]. Results thus suggested that the N-fertilization of plant under high CO_2_ was able to enhance the soluble sugar and starch contents, which had simultaneously enhanced the TNC. Similar observation was found by other researchers [20,21,22,23]. The accumulation of carbohydrate in low nitrogen-fertilized plant might be due to the reduction in sink size of the plant when nitrogen is limited; hence, reducing the translocation of carbohydrates to other plant parts [24]. When sink strength was reduced under low nitrogen fertilization, the extra carbohydrates accumulated in *L. pumila* plants might be channeled for the production of secondary metabolites (total phenols and flavonoids), thus explaining the reason why the production of secondary metabolites was up-regulated in low nitrogen fertilization. It is possible that when photosynthetic performance is suppressed under insufficient nitrogen supply, recycling of the enzymatic nitrogen required for secondary metabolism may occur resulting in possible increase in secondary metabolites (phenolics and flavonoids) [25].

**Table 3:** Accumulation and partitioning of total soluble sugar (TSS), starch and total non structural carbohydrate (TNC) in different plant parts of *Labisia pumila* Benth. under different combinations of nitrogen levels and CO_2_ enrichment.

Nitrogen levels	Plant parts	TSS (mg sucrose /g dry weight)	Starch (mg glucose /g dry weight)	TNC (mg /g dry weight)
A -180 kg N ha^-1^*	Leaf	24.06 ±0.51c	60.11 ± 0.19d	84.16 ± 2.32c
	Stem	16.70 ± 1.15d	23.01 ± 0.27d	40.14 ± 1.67d
	Root	20.21 ± 0.51c	49. 6 ± 0.33d	70.21 ± 2.32c
0 kg N ha^-1^	Leaf	42.71 ± 0.50a	89.41 ± 0.28a	131.32 ± 3.21a
	Stem	32.04 ± 0.84a	80.16 ± 0.29a	113.21 ± 1.56a
	Root	38.09 ± 0.50a	78.22 ± 0.49a	117.34 ± 3.56a
90 kg N ha^-1^	Leaf	37.45 ± 0.49b	78.18 ± 0.62b	116.7 ± 5.77b
	Stem	22.26 ± 0.44b	45.27 ± 0.47b	67.23 ± 6.22b
	Root	33.66 ± 0.49b	67.98 ± 0.51b	103.21 ± 7.55b
180 kg N ha^-1^	Leaf	32.64 ± 0.59c	86.22 ± 0.23c	118.78 ± 5.67b
	Stem	15.10 ± 0.99d	51.03 ± 0.25c	66.31 ± 6.90c
	Root	28.86 ± 0.58c	66.67 ± 0.44c	96.75 ± 7.90c
270 kg N ha^-1^	Leaf	23.80 ± 1.16d	57.66 ± 0.23d	82.22 ± 6.89d
	Stem	8.96 ± 0.70d	32.42 ± 0.21d	41.31 ± 8.65d
	Root	20.02 ± 1.17d	48.20 ± 0.28d	68.31 ± 7.96d

All analyses are mean ± standard error of mean (SEM), N = 15. Means not sharing a single letter were significantly different at P ≤ 0.05. A = Control at ambient CO_2_ levels (400 µmol^-1^ mol^-1^) and standard Nitrogen fertilization rates (180 kg N ha^-1^).

**Table 4:** Effects of different nitrogen levels on some physiological parameters in *L. pumila* Benth. under CO_2_ enrichment.

Parameters	*Ambient-180 kg N h^-1^	0 kg N h^-1^	90 kg N h^-1^	180 kg N h^-1^	270 kg N h^-1^
Photosynthesis	5.29±0.04c	4.98 ± 0.25a	7.29 ± 0.26b	8.46 ± 0.24c	11.75 ± 0.47d
PNUE^1^	1.79 ± 0.06c	3.09 ± 0.18a	3.01 ± 0.17b	2.45 ± 0.12c	2.66 ± 0.12d
Leaf N content	2.96 ± 0.10c	1.63 ± 0.09a	2.46 ± 0.11b	3.47 ± 0.09c	4.42 ± 0.08d
C:N^2^	12.64 ± 0.65c	28.15 ± 1.15a	18.29 ± 0.72b	13.12 ± 0.42c	10.29 ± 0.18d
Chlorophyll a	5.48 ± 0.06c	3.60 ± 0.123a	4.35 ± 0.123b	5.30 ± 0.05c	6.16 ± 0.10d
Chlorophyll b	16.38 ± 0.15c	12.30 ± 0.26a	13.92 ± 0.26b	15.96 ± 0.11c	17.85 ± 0.22d
Chlorophyll a +b	21.86 ± 0.21c	15.90 ± 0.39a	18.21 ± 0.38b	21.26 ± 0.16c	24.01 ± 0.33d
Total soluble sugar	24.04 ± 0.52c	42.72 ± 0.50a	37.45 ± 0.49b	32.64 ± 0.60c	23.80 ± 1.17d
Starch	58.04 ± 0.52c	84.05 ± 2.11a	78.78 ± 2.18b	72.75 ± 2.41c	63.91 ± 2.98d
TNC^3^	82.07 ± 1.03c	126.77 ± 2.47a	116.24 ± 2.58b	105.40 ± 2.93c	87.71 ± 4.10d
Total flavonoids	0.31 ± 0.01c	0.51 ± 0.01a	0.47 ± 0.01b	0.33 ± 0.01c	0.30 ± 0.01d
Total phenolics	1.16 ± 0.21c	1.43 ± 0.14a	1.35 ± 0.23b	1.25 ± 0.32c	0.71 ± 0.24d

All analyses are mean ± standard error of mean (SEM), N = 15. Means not sharing single letter were significantly different at P ≤ 0.05. *Control at ambient CO_2_ levels (400 µmol^-1^ mol^-1^) and standard nitrogen fertilization rate (180 kg N ha^-1^). ^1^ = Photosynthesis nitrogen use efficiency (µmol mol^-1^ N s^-1^); ^2^ = carbon to nitrogen ratio; ^3^ = total non structural carbohydrate (mg g^-1^).

### 2.3. Photosynthesis and Photosynthesis Nitrogen Use Efficiency (PNUE)

The net assimilation rate (photosynthesis) was influenced by nitrogen levels applied (*P* ≤ 0.05), however, no varietal differences were observed. Leaf photosynthesis rate increased with increasing nitrogen fertilization in an ascending oder 0 > 90 > 180 > 270 kg N ha^-1^. The highest photosynthesis was obtained in *L. pumila* exposed to CO_2_ enrichment combined with 270 kg N ha^-1^ (11.75 µmol m^-2^ s^-1^) compared to without N fertilization (4.98 11.75 µmol m^-2^ s^-1^; Table 4). However, under ambient CO_2_, plants recorded 6% higher photosynthesis (5.29 µmol m^-2^ s^-1^) when fertilized with 180 kg N ha^-1^ compared to the plant raised under elevated CO_2_ (4.98 µmol m^-2^ s^-1^) but without N fertilization (0 kg N Ha^-1^). The finding showed the importance N in further enhancing leaf gas exchange of *L. pumila* plants exposed to CO_2_ enrichment. 

Plants fertilized with less N levels were inclined to record higher PNUE values. The increase in PNUE signified that the plants were more efficient in utilizing nitrogen due low nitrogen availability [26]. Results of the present study showed that a decrease in photosynthesis could have stimulated the production of plant secondary metabolites, as shown by the negative correlation coefficient (Table 2) between photosynthesis and secondary metabolites (r^2^ = -0.77) of total phenolics and flavonoids. A possible explanation to this might be that the decrease in photosynthetic rate could have increased the shikimic acid pathway that enhanced the production of plant secondary metabolites, and this is due to increase in the concentration of soluble sugar [27].

### 2.4. Leaf Nitrogen and Carbon to Nitrogen Ratio (C:N)

The enhancement of N fertilization significantly improved leaf nitrogen content (*P* ≤ 0.05). As nitrogen levels increased from 0 to 270 kg N ha^-1^ leaf tissue nitrogen also increased considerably. The increase in leaf tissue nitrogen might result from intensification of nitrate content in the leaf that signified the enhanced nitrate assimilation of plant under elevated CO_2_ [28]. Simultaneously, the increase in leaf nitrogen content had lead to reduction in plant C:N ratio under high N fertilization. High CO_2_ treatment combined with the highest nitrogen level (270 kg N ha^-1^) reduced the C:N ratio (10.29), whilst when combined with 0 kg N ha^-1^, had increased the C:N ratio (28.15) by 173%. A similar increase in C:N ratio of plants enriched with high CO_2_ under low nitrogen was also observed by Fonseca *et al*. [23]. High C:N ratio had a significant positive relationship (*P* ≤ 0.01) with total flavonoids and phenolics compounds (r^2^ = 0.81; Table 2) signifying a good direct association between the C:N ratio and plant secondary metabolites. Conversely, the C:N ratio displayed a significant negative relationship with photosynthesis (r^2^ = -0.71), implying that increase in C:N ratio decreased the photosynthetic capacity of *L. pumila*. Winger et al. [29] attributed the increase in C:N ratio that had decreased the photosynthetic capacity to increase in carbohydrate accumulation, which repressed photosynthetic protein production, especially the Rubisco. In the present study, the increase in C:N ratio had also reduced the photosynthetic capacity of *L. pumila* seedlings, and this suggested an enhanced synthesis of plant secondary metabolites, especially the flavonoids and phenolics [30].

### 2.5. Chlorophyll Content

Chlorophyll content was influenced by the application of Nitrogen (*P* ≤ 0.01). As the levels of N fertilization increased from 0 to 270 kg N ha^-1^, chlorophyll a, b and total chlorophyll a+b were also enhanced. The increase in chlorophyll content with increasing nitrogen has been reported by Suza and Valio [31]. It was found from the correlation (Table 2) that chlorophyll a, b and total were significantly (*P* ≤ 0.01) and negatively related. Competition between secondary metabolites and chlorophyll content fits well with the prediction of protein competition model (PCM) that the secondary metabolites content is controlled by the competition between protein and secondary metabolites biosynthesis pathway and its metabolites regulation. The negative relationship between the secondary metabolites and chlorophyll is a sign of gradual switch of investment from protein to polyphenols production [32]. The same discovery was also obtained by Michel *et al.* [33] on flavonoids and chlorophyll content in *Arabidopsis*, which suggested that the production of secondary metabolites was competing with light harvesting protein when soil nitrogen was low.

## 3. Experimental

### 3.1. Experimental Location, Plant Materials and Treatments

The experiments were carried out under a growth house at Ladang 2, Faculty of Agriculture Glasshouse Complex, Universiti Putra Malaysia (longitude 101° 44’ N and latitude 2° 58’S, 68 m above sea level) with a mean atmospheric pressure of 1.013 kPa. Three-month old *L. pumila* seedlings of var *alata,* var *pumila* and var *lanceolata* were left for a month to acclimatize in a nursery until ready for the treatments. Carbon dioxide enrichment treatment started when the seedlings reached 4 months of age where plants were exposed to 1,200 µmol^-1^ mol^-1^ CO_2_ and fertilized with four levels of nitrogen concentrations viz. 0, 90, 180 and 270 kg N Ha^-1^. The fertilization with nitrogen levels were split into three applications. A control at ambient CO_2_ (400 µmol^-1^ mol^-1^) with standard N fertilization (180 kg N ha^-1^) was included to compare plant responses to high CO_2_ combined with different levels of N. This factorial experiment was arranged in a split plot using a randomized complete block design with nitrogen levels being the main plot, and varieties as the sub-plot replicated three times. Each treatment consisted of ten seedlings. 

### 3.2. Growth House Microclimate and CO_2_ Enrichment Treatment

The seedlings were raised in specially constructed growth houses receiving 12-h photoperiod and average photosynthetic photon flux density of 300 µmol m^-2^ s^-1^. Day and night temperatures were recorded at 30 ± 1.0 °C and 20 ± 1.5 °C, respectively, and relative humidity at about 70% to 80%. Vapor pressure deficit ranged from 1.01 to 2.52 kPa. Carbon dioxide at 99.8% purity was supplied from a high–pressure CO_2_ cylinder and injected through a pressure regulator into fully sealed 2 m × 3 m growth houses at 2-h daily and applied continuous from 08:00 to 10:00 a.m. [34]. The CO_2_ concentration at different treatments was measured using Air Sense ™ CO_2_ sensors designated to each chamber during CO_2_ exposition period. Plants were watered three to four times a day at 5 min per session to ensure normal growth of plant using drip irrigation with emitter capacity of 2 L hr^-1^. The experiment lasted for 15 weeks from the onset of treatment.

### 3.3. Total Phenolics and Total Flavonoid Quantification

The method of extraction and quantification for total phenolics and flavonoids contents followed after Jaafar *et al.* [8]. An amount of ground tissue sample (0.1 g) was extracted with 80% ethanol (10 mL) on an orbital shaker for 120 minutes at 50 °C. The mixture was subsequently filtered (Whatman™ No.1), and the filtrate was used for the quantification of total phenolics and total flavonoids. Folin – Ciocalteu reagent (diluted 10-fold) was used to determine the total phenolics content of the leaf samples. Two hundred µl of the sample extract was mixed with Follin–Ciocalteau reagent (1.5 mL) and allowed to stand at 22 °C for 5 minutes before adding NaNO_3_ solution (1.5 mL, 60 g L^-1^). After two hours at 22 °C, absorbance was measured at 725 nm. The results were expressed as mg g^-1^ gallic acid equivalent (mg GAE/ g dry sample). For total flavonoids determination, sample (1 mL) was mixed with NaNO_3_ (0.3 mL) in a test tube covered with aluminium foil, and left for 5 minutes. Then 10% AlCl_3_ (0.3 mL) was added followed by addition of 1 M NaOH (2 mL) and the absorbance was measured at 510 nm using rutin as a standard (mg rutin/ g dry sample). 

### 3.4. Starch Determination

Starch content was determined spectrophometrically using method by Thayumanavam and Sadasivam [35]. In this method, dry sample (about 0.5 g) was homogenized in hot 10 ml 80% ethanol to remove the sugar. The sample was then centrifuged at 5,000 rpm for 5 minute and then the residue was retained. After that, distilled water (5.0 mL) and 52% perchloric acid (6.5 mL) were added to the residue, then the solution was centrifuged and the supernatant separated and then filtered through no. 5 filter paper (Whatman). The processes were repeated until the supernatant was made up to 100 mL. An aliquot of the supernatant (100 µL) was added to distilled water until the volume became 1 mL. After that, 4 ml anthrone reagent (Sigma, USA; prepared with 95% sulphuric acid by adding 2 g of anthrone to 100 ml 95% sulphuric acid) was added to a tube. The mixed solution was placed in the water bath at 100 °C for eight minutes and then cooled to the temperature room, and then the sample was read at absorbance of 630 nm to determine the sample starch content. Glucose was used as a standard and starch content was expressed as mg glucose equivalent /g dry sample. 

### 3.5. Soluble Carbohydrates

Soluble carbohydrates were measured spectrophotometrically using the method described by Edward [36]. Samples (0.5 g) were placed in 15 mL conical tubes. Then distilled water (10 mL) was added and the mixture was then vortexed and incubated for 10 minutes. Anthrone reagent was prepared using anthrone (0.1 g) that was dissolved in 95% sulphuric acid (50 mL). Sucrose was used as a standard stock solution to prepare a standard curve for the quantification of sucrose in the sample. The mixed sample of ground dry sample and distilled water was centrifuged at a speed of 3,400 rpm for 10 minutes and then filtered to get the supernatant. To an aliquot ( 4 mL) of the sample was added anthrone reagent (8 mL) and the mixture was placed in a waterbath set at 100 °C for 5 minutes before the sample was measured at absorbance 620 nm using UV160U spectrophotometer (Shimadzu, Japan). The soluble sugar in the sample was expresses as mg sucrose/ g dry sample.

### 3.6. Total Non Structural Carbohydrate (TNC)

The total non structural carbohydrate was calculated as the sum of total soluble sugar and starch content [24].

### 3.7. Chlorophyll Content

Total chlorophyll content was measured by method from Idso *et al.* [37] using fresh weight basis. Prior to each destructive harvest each seedling was analyzed for the leaf chlorophyll relative reading (SPAD meter 502, Minolta Inc, USA). The leaves of *Labisia pumila* with different greenness (yellow, light green and dark green) were selected for analysis and total leaf chlorophyll content was analyzed. For each type of leaf greenness, the relative SPAD value was recorded (five points/leaf) and the same leaves sampled for chlorophyll content determination. Leaf disk 3 mm in diameter was obtained from leaf sample using a hole puncher. For each seedling the measurement was conducted on the youngest fully expanded leaves on each plant, generally the second or third leaf from the tip of the stem was used. The leaf disks were immediately immersed in acetone (20 mL) in an aluminum foil-covered glass bottle for approximately 24 hours at 0 °C until all the green colour had bleached out. Finally, the solution (3.5 mL) was transferred to measure at absorbances of 664 and 647 nm using a spectrometer (UV-3101P, Labomed Inc, USA). After that the least squares regression was used to develop predictive relation between SPAD meter readings and pigment concentrations (mg / g fresh weight) obtained from the chlorophyll destructive analysis.

### 3.8. Total Carbon, Nitrogen and C:N Ratio

Total carbon and C:N ratio were measured by using a CNS 2000 analyzer (Model A Analyst 300, LECO Inc, USA). This was performed by placing ground leaf sample (0.05 g) into the combustion boat. Successively, the combustion boat was transferred to the loader before the sample was burned at 1,350 °C to obtain the reading of total carbon and nitrogen content of the samples. 

### 3.9. Photosynthesis and Photosynthesis Nitrogen Use Efficiency (PNUE)

The measurement was obtained from a closed infra-red gas analyzer LICOR 6400 Portable Photosynthesis System system (IRGA, Licor Inc. Nebraska, USA). Prior to use, the instrument was warmed for 30 minutes and calibrated with the ZERO IRGA mode. Two steps are required in the calibration process: first, the initial zeroing process for the built-in flow meter; and second, zeroing process for the infra-red gas analyzer. The measurements used optimal conditions set by Jaafar et al. [38] of 400 µmol/mol CO_2_ 30 °C cuvette temperature, 60% relative humidity with air flow rate set at 500 cm^3^/min, and modified cuvette condition of 800 µmol m^-2^ s^-1^ photosynthetically photon flux density (PPFD). The measurements of gas exchange were carried out between 09:00 to 11:00 a.m. using fully expanded young leaves numbered three and four from plant apex to record net photosynthesis rate (A). The operation was automatic and the data were stored in the LI-6400 console and analyzed by “ *Photosyn Assistant*“ software (Version 3, Lincoln Inc, USA) [38] Several precautions were taken to avoid errors during measurements. Leaf surfaces were cleaned and dried using tissue paper before enclosed in the leaf cuvette. Photosynthesis nitrogen use efficiency (PNUE) was calculated by dividing the photosynthesis to nitrogen content of leaves [39].

### 3.10. Statistical Analysis

Data were analyzed using analysis of variance by SAS version 17. Mean separation test between treatments was performed using Duncan multiple range test and standard error of differences between means was calculated with the assumption that data were normally distributed and equally replicated.

## 4. Conclusions

This study demonstrated that enrichment with high levels of CO_2_ can enhance the production of plant secondary metabolites, in particular the total phenolics and total flavonoids, in plant. However, increased nitrogen fertilization can reduce the production of these plant secondary metabolites under CO_2_ enrichment. When there were accumulation of TNC in plant leaves and reduction in photosynthesis, the production of plant secondary metabolites might be up-regulated. The increase in the production of plant secondary metabolites was indicated by increases in the values of C:N ratio, PNUE and reduction in chlorophyll contents.
